# Targeting G-quadruplex by TMPyP4 for inhibition of colorectal cancer through cell cycle arrest and boosting anti-tumor immunity

**DOI:** 10.1038/s41419-024-07215-2

**Published:** 2024-11-11

**Authors:** Peisi Li, Dawang Zhou, Yumo Xie, Ze Yuan, Mingzhe Huang, Gaopo Xu, Junfeng Huang, Zhuokai Zhuang, Yanxin Luo, Huichuan Yu, Xiaolin Wang

**Affiliations:** 1 Guangdong Institute of Gastroenterology, Guangdong Provincial Key Laboratory of Colorectal and Pelvic Floor Disease, The Sixth Affiliated Hospital, Sun Yat-sen University, Guangzhou, Guangdong China; 2 Biomedical Innovation Center, The Sixth Affiliated Hospital, Sun Yat-sen University, Guangzhou, Guangdong China; 3 Department of Hepatobiliary and Pancreatic Surgery, Sun Yat-Sen University Cancer Center, Guangzhou, Guangdong China; 4 Department of General Surgery (Colorectal Surgery), The Sixth Affiliated Hospital, Sun Yat-sen University, Guangzhou, Guangdong China

**Keywords:** Cancer microenvironment, Cancer therapy

## Abstract

G-quadruplex (G4) is a noncanonical DNA secondary structure known to induce DNA damage and regulate the expression of immune-related genes. We aim to exploit the G4 folding as a treatment strategy to trigger anti-tumor immune response. In this study, we observe that the abundant genomic G4 in epithelial cells coexists with increased infiltration of CD8^+^ T cells in colorectal cancer tissue. Furthermore, our data substantiate the inhibitory effect of the G4 ligand TMPyP4 on cancer progression while concurrently stimulating anti-tumor immunity. Mechanistically, TMPyP4 impedes cancer cell proliferation and induces G2/M cell cycle arrest. Additionally, in vivo experiments demonstrate that TMPyP4 enhances the anti-tumor immune response by triggering DNA damage and activating the cGAS-STING pathway, which fosters CD8^+^ T cell activation and dendritic cell maturation. Importantly, the combined treatment of TMPyP4 and anti-PD1 exhibits a synergistic therapeutic effect on colorectal cancer. In summary, our findings underscore the potential of the G4 ligand TMPyP4 as a dual strategy to target colorectal cancer: inhibiting cancer progression and augmenting anti-tumor immunity through the activation of cGAS-STING pathway.

## Highlights

- The tissue region with abundant G4 in epithelial cells exhibits more infiltration of CD8^+^ T cells in colorectal cancer.
- The G4 ligand TMPyP4 inhibits cancer cell proliferation by inducing a G2/M cell cycle arrest.
- The G4 ligand TMPyP4 promotes the activation of CD8^+^ T cells and the maturation of DCs by activating the cGAS-STING pathway.

## Introduction

The landscape of cancer treatment has witnessed a transformative shift with the advent of immunotherapies, notably the programmed cell death 1 (PD-1)/PD-1 ligand (PD-L1) checkpoint blockade [1, 2]. This approach induces durable responses by triggering long-term antitumor immunity [3]. However, only a limited proportion of patients respond to current immunotherapy [4–6]. Recent evidence highlights the critical role of T lymphocyte infiltration, particularly CD8^+^ T cells, in determining responses to immunotherapy in various cancers, including colorectal cancer (CRC) [7–9].

In CRC, the intratumoral infiltration of CD8^+^ T cells is associated with favorable clinical outcomes [10]. Chemokines, through their interaction with corresponding receptors, play pivotal roles in controlling immune trafficking and tumor infiltration. Specifically, CCL5 and CXCL10, which are generated at the tumor site, are crucial in determining the optimal infiltration of CD8^+^ T cells [11, 12]. Understanding these mechanisms is essential for enhancing immunotherapy responsiveness in CRC.

Guanine-rich DNA sequences can fold into unique four-stranded G-quadruplex (G4) structures. These structures form through the alignment of four G bases held together by Hoogsteen hydrogen bonds and coordinated by metal ions, typically K^+^ [13]. The distribution of these putative G4 structures within the nuclear genome isn’t random; they tend to cluster in specific regions such as promoters, ribosomal DNA, and telomeres [14]. Recent investigations have revealed that modulating G4 structures with G4-interactive binding ligands can exacerbate replicative stress by disrupting essential DNA transactions such as replication and transcription, resulting in single- and double-stranded breaks and activation of the DNA damage response (DDR) machinery [15]. Therefore, promoting G4 formation holds potential in cancer therapeutics. Moreover, this phenomenon serves as a source of cytoplasmic DNA, subsequently triggering the expression of type I interferon (IFN) genes [16]. These IFN genes and related pathways have been proven to enhance anti-tumor CD8^+^ T cells immunity [17]. Nevertheless, the specific involvement and the underlying mechanisms of G4 in CD8^+^ T cell infiltration have remained to be elucidated.

Therefore, we aimed to explore the value and mechanism of G4-targeting strategy with G4 ligand in boosting cancer treatment and anti-tumor immune response. Our study found a positive correlation between G4 density and the presence of CD8^+^ T cells, as well as improved patient survival in CRC. It was revealed that the G4 ligand TMPyP4 enhances the activation of CD8^+^ T cells and DCs within tumors by activating the cGAS-STING pathway. Furthermore, we demonstrated that TMPyP4 could sensitize anti-PD1 treatment. The combined treatment with TMPyP4 and immunotherapy could serve as a promising regimen for CRC.

## Materials and Methods

### Multiplex immunohistochemistry (mIHC)

The expression intensity and spatial distribution of G4, CD8, PanCK, CD11b, and P-STING in tissues were labeled using mIHC. The tissues were fixed in formalin and then dehydrated and embedded in paraffin. The paraffin block containing tissues was cut into sections. FFPE tissue slides were melted and dehydrated at 60 °C and then deparaffinized and rehydrated using xylene and alcohol. The paraffin slides were placed in a microwave oven in citrate buffer (pH 6.0) for heat-induced antigen retrieval. The blocking buffer (X0909; Dako, Santa Clara, CA, USA) was used to block the sections for 30 min. Supplemental Table 1 shows the antibodies used for the staining and their manufacturers. The slides were incubated with the primary antibody and horseradish peroxidase-conjugated secondary antibody, and tyramine signal amplification (TSA) was performed. Antibody stripping and antigen retrieval were performed after each round of TSA. Then, DAPI was used to stain nuclei. The antibodies are shown in Supplemental Table S1.

### Immunohistochemistry

In brief, antigen retrieval was performed by boiling slides in a citrate solution. FFPE tissue slides were blocked with blocking buffer and with a primary antibody overnight and then incubated with peroxidase-conjugated secondary antibodies. The DAB (Zhong Shan Jin Qiao) was used for the antigens stained. IHC scores were determined by the intensity score and the proportion of area positively stained tumor cells. The final score was the average of three cores from the representative tumor area. The antibodies are shown in Supplemental Table S1.

### Cell Lines and Drug Treatment

The DLD1, HCT8, HCT15, LOVO, SW620, CACO2, and CT26 were maintained in RMPI 1640 medium. The HCT116, LST174T, RKO, NCI-H508, and MC38 were cultured in high-glucose Dulbecco’s Modified Eagle Medium. All cell culture medium was supplemented with 10% FBS and 1% penicillin/streptomycin. All cell lines were confirmed negative for mycoplasma by PCR-based method. TMPyP4 was purchased from MCE (HY-108477). For the colony formation assay, single-cell suspensions were plated (1000 cells per well) in 6-well plates, and the medium was refreshed every three days for nine days of culture. Colonies were fixed in methanol, stained with 0.5% crystal violet, and photographed. For the cell viability assay, cells were seeded in 96-well plates. After 8 h, cells were treated with different concentrations of drugs and cultured at 37 °C for 72 h, and the number of viable cells was measured using the counting kit-8 (APExBIO). For the apoptosis assay, Annexin V-FITC/PI Apoptosis Kit (AP101) was used to analyze apoptosis. For the cell cycle assay, cells were treated with RNase A and PI for 30 min, and the distribution of cells in the G0/G1, S, and G2/M phases was examined by flow cytometry.

### Cell infections and transfections

The target plasmid used to express the shRNAs targeting mouse STING was purchased from IGEA. 293 T cells were co-transfected with the target plasmid and packaging plasmids psPAX2 and pMD2.G at 4:3:1. The viral supernatant was collected post-transfection, filtered, and added to target cells. And the tumor cells were selected with the antibiotic. The efficiency was validated by immunoblotting.

### Animal experiments

Four to six-week-old female C57BL/6 mice, male BALB/c, and nude mice were purchased from GemPharmatech (China). Mice were maintained at a specific pathogen-free facility with a 12 h/12 h day/night turnover. All animal experiments were conducted in accordance with the National Institutes of Health Guide for the Care and Use of Laboratory Animals and were approved by the Research Ethical Committee of the Sixth Affiliated Hospital of Sun Yat-sen University. For the pharmaceutical experiments of TMPyP4, mice of similar tumor burden were divided into two groups: treatment with vehicle and 30 mg/kg TMPyP4 (three times a week, i.p.). For the establishment of a PDX model, Nude mice were implanted subcutaneously with pieces of fresh tumor from CRC patients in surgery. Tumor-bearing mice with similar tumor burdens were randomly divided into control and experimental groups for drug treatment. Tumors were excised either upon reaching 1500 mm^3^ or at the end of the study. For estimation of immunotherapy efficacy, randomization was performed by equally dividing tumor‐bearing mice of similar tumor burden into control and drug treatment groups: treatment with vehicle, TMPyP4 (three times a week, i.p.), anti-PD1 (three times a week, i.p.), and a combination in which each compound was administered at the same dose and schedule as the single agent.

### Lymphocyte staining and flow cytometry

Anti-CD16/32 antibodies (eBioscience) were incubated with tumor single cells on ice for 20 min. For surface staining, cells were exposed to surface marker antibodies for 30 min and then washed in FACS buffer. Flow cytometry directly analyzed the cells. For intracellular cytokine staining, cells were stimulated with ionomycin (MCE) and phorbol 12-myristate13-acetate (MCE) for 6 h in the presence of monensin (MCE) and Brefeldin A (MCE). After surface molecule staining, a fixation/permeabilization buffer solution was used, following the manufacturer’s protocol (ThermoFisher). The antibodies are shown in Supplemental Table 1.

### Hemolysis assay

Different concentrations of TMPyP4 were prepared by diluting them in a centrifuge tube. From each tube, 100 μl of the diluted TMPyP4 was transferred and incubated with 100 μl of freshly washed murine red blood cells at 37 °C for 1 h. Triton X-100 (Sigma-Aldrich, USA) served as the positive control. Subsequently, the mixture was centrifuged at 3000 rpm for 15 min. A volume of 100 μL of the supernatant was then transferred to a new 96-well plate. The absorbance at 540 nm was measured using the Varioskan Flash (Thermo Scientific). The percentage of hemolysis was calculated by comparing the results to the positive control (100% hemolysis), with the optical density (OD) values adjusted to account for background interference.

### Quantitative PCR analysis

Cells were subjected to a Trizol-Based Method for total RNA isolation. The retrotranscription of RNA was carried out using the HiScript III-RT SuperMix for qPCR (+gDNA wiper) (Vazyme). Quantitative PCR (qPCR) was conducted on cDNA with ChamQ Universal SYBR qPCR Master Mix (Vazyme). The ΔΔCT method, employing GAPDH or ACTB as an endogenous control, was utilized to calculate mRNA fold changes. Results were normalized to the controls and expressed as fold changes. The primers for gene expression analysis can be found in Supplemental Table S2.

### Western blot analysis

Following cold PBS washing, cell lysis was carried out using cold RIPA lysis buffer (Beyotime, P0013B) supplemented with 1× protease and phosphatase inhibitor cocktail (MCE). The lysis process involved slow shaking on ice for 30 min, followed by centrifugation at 15,000 g for 20 min at 4 °C. Protein concentration was determined using a BCA protein assay kit (ThermoFisher), and subsequent denaturation occurred at 98 °C for 5 min. Separation of 10 μg total protein was achieved through SDS-PAGE, and transfer to a PVDF membrane (Millipore) ensued.

After pre-incubation with 5% w/v bovine serum albumin (BSA) for 1 h, the PVDF membranes were subjected to overnight incubation with primary antibodies at 4 °C. The following day, hybridization with an HRP-conjugated secondary antibody (Promega) was performed for 1 h at room temperature. Protein bands were visualized using Western ECL Blotting Substrates (Bio-Rad) and captured using the ChemiDoc^TM^ Imaging System (Bio-Rad). The antibodies are shown in Supplemental Table S1.

### Statistical Analysis

GraphPad Prism 7.0 was used for statistical analysis. Statistical significance, with a threshold set at P < 0.05, is represented in the data as mean ± SD. To compare the two groups, a two-tailed unpaired t-test was employed. For analyses involving multiple groups, group differences were assessed using either one-way or two-way ANOVA. Subsequent multiple comparisons were conducted using Tukey’s or Bonferroni tests as appropriate.

## Results

### G4 positively correlates with the density of CD8^+^ T cells and better patient survival

**Fig. 1:**
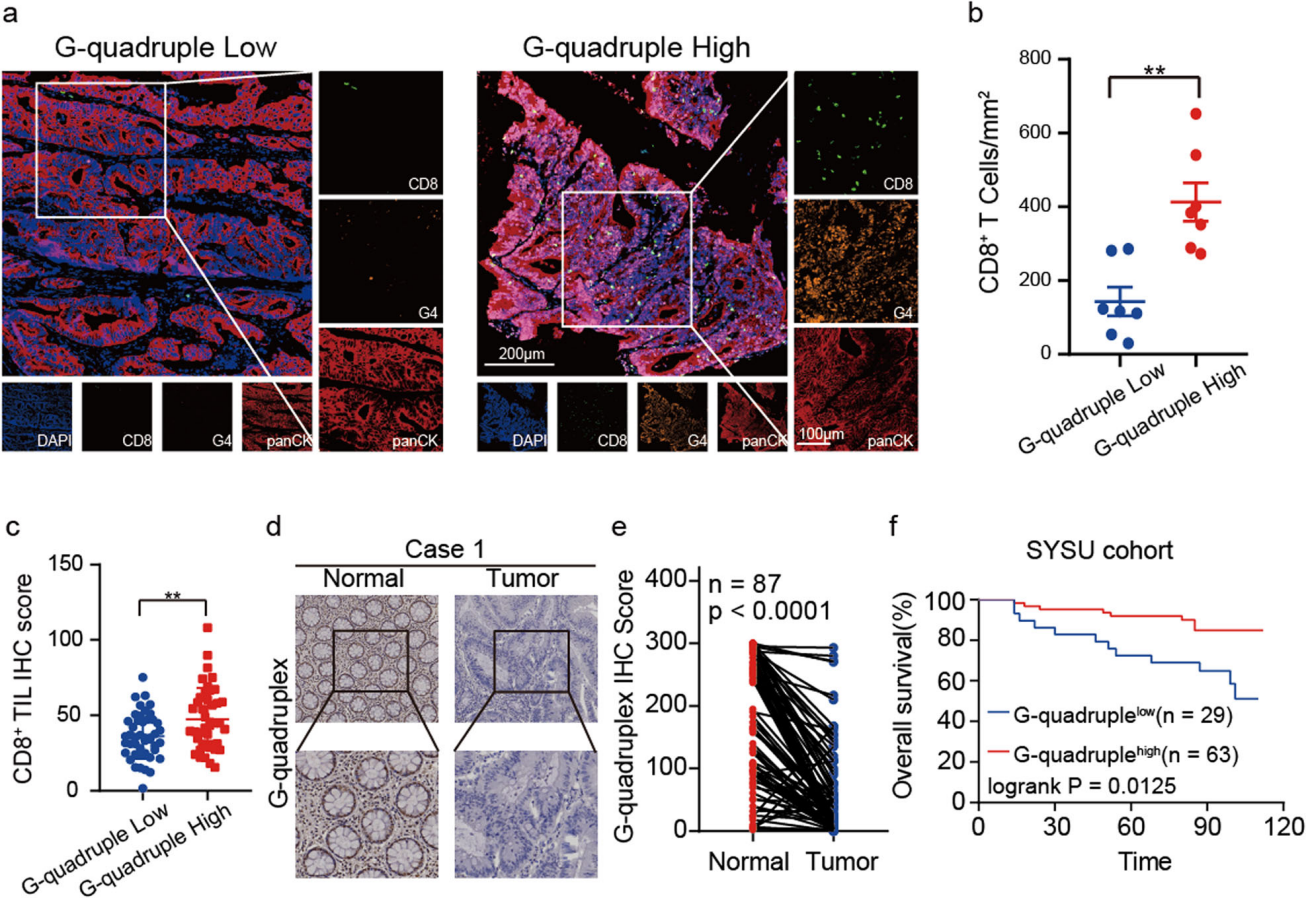
G4 positively correlates with the density of CD8^+^ T cells and patient survival in CRC. **a** Representative immunostainings of CD8, G4, and panCK in a colorectal cancer case, highlighting higher G4 expression compared to a case with lower G4 expression (CD8 [green], G4 [orange], PANCK [red], DAPI [blue]) (magnification ×200). **b** Correlation between G4 and CD8 expression in a. ^**^p ≤ 0.01, by unpaired t-test. **c** CD8^+^ T cell score was higher in patients with high G4 expression by IHC. ^**^p ≤ 0.01, by unpaired t-test. **d** Representative images of G4 IHC staining. **e** Statistical analysis of G4 levels of 87 paired samples of CRC and adjacent normal tissues from the Sixth Affiliated Hospital. ^****^p ≤ 0.0001, by paired t-test. **f** For Kaplan-Meier survival analysis, the lower tertile of G4 expression was selected as the cutoff to split high and low expresser populations.

Analysis of G4 and CD8^+^ T cells in CRC samples, utilizing multiplex immunohistochemistry (mIHC), unveiled a higher abundance of CD8^+^ T cells in tumors characterized by elevated G4 expression (Fig. 1a, b). This association was consistently observed in immunohistochemistry (IHC) analyses, confirming an augmented presence of CD8^+^ T cells in G4 high-expressing tumors (Fig. 1c). Moreover, our investigation revealed that G4 expression is present across all layers of normal tissue, including the mucosa, submucosa, and muscularis propria (Supplementary Fig. 1a). Additionally, we observed a downregulation of G4 expression in tumor tissues compared to non-tumor counterparts (Fig. 1d, e). This downregulation was consistent across both human and murine cell lines, highlighting a significant decrease in G4 expression in cancer cells compared to normal cells (Supplementary Fig. 1b). Furthermore, Kaplan-Meier survival curves emphasized poorer outcomes for cases with low G4 expression (Fig. 1f). Collectively, these findings indicate that higher G4 expression is associated with increased CD8^+^ T cells and improved patient survival.

### G4 ligand TMPyP4 inhibits the development and progression of colorectal cancer

**Fig. 2:**
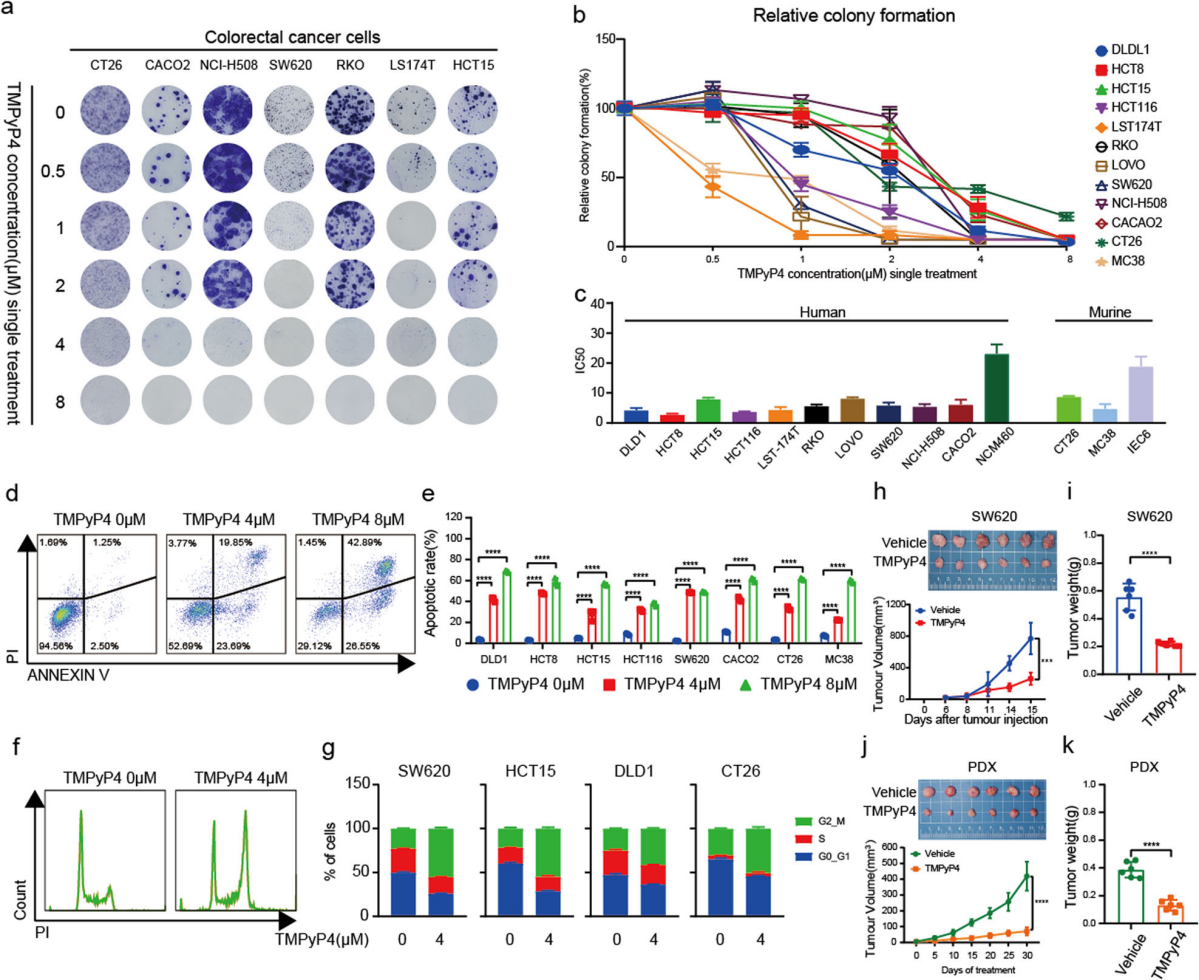
G4 ligand TMPyP4 hinders the development and progression of CRC. **a** Representative images of colony formation in multiple human colorectal cancer cell lines HCT15, LST174T, RKO, SW620, NCI-H508, Caco2, and mouse colon cell line CT26 treated with the indicated concentrations of TMPyP4. **b** The line graph represents the relative colony formation of colorectal cancer cells treated with the indicated concentrations of TMPyP4. **c** Cell viability of colorectal cancer cells and normal cells was measured with the CCK8 assay. Cells were treated with TMPyP4 for 72 h. **d**, **e** Cells were treated with 4 μM or 8 μM TMPyP4 for 48 h. Then, the proportion of apoptotic cells was detected using annexin V-propidium iodide-based flow cytometry. Values are represented as mean ± SD, ^****^p ≤ 0.0001, by multiple t-tests. **f**, **g** Cell cycle analysis of colorectal cancer cells treated with or without TMPyP4 for 24 h. Cellular DNA content was determined by propidium iodide staining and flow cytometry. **h, i** The tumor size (**h**) and tumor weight (**i**) of nude mice bearing SW620 colorectal tumors treated with vehicle or 30 mg/kg TMPyP4. *n* = 6 mice for both groups. Values are represented as mean ± SD, ^****^p ≤ 0.0001, by unpaired t-test. **j**, **k** The tumor size (**j**) and tumor weight (**k**) of nude mice treated with vehicle or 30 mg/kg TMPyP4 in the PDX model. *n* = 6 mice for both groups. Values are represented as mean ± SD, ^****^p ≤ 0.0001, by two-way ANOVA (**h**, **j**) or unpaired t-test (**i**, **k**).

Although the G4 ligands, such as Pyridostatin (PDS), BRACO-19 (B19), and 5, 10, 15, 20-tetrakis (1-methylpyridinium-4-yl) porphyrin (TMPyP4), are well-known, their specific abilities to induce G4 formation in colorectal cancer cells are still unclear [18–20]. Our investigation highlighted TMPyP4’s superior capability in inducing G4 structures, as evidenced by flow cytometry analysis (Supplementary Fig. 2a, b) and immunofluorescence assay in the SW620 cell line (Supplementary Fig. 2c). Consequently, TMPyP4 was selected as the G4 ligand for the subsequent phase of our study. Systematic analysis of TMPyP4 sensitivity, utilizing colony assay formation with human and murine cell lines, revealed a significant reduction in colony formation across all tested colorectal cell lines (Fig. 2a, b). Moreover, TMPyP4 concentration-dependently decreased the viability of colorectal cell lines, while demonstrating weak effects on normal cell lines NCM460 and IEC6 (Fig. 2c). Notably, TMPyP4 induced a significant increase in apoptosis in CRC cell lines (Fig. 2d, e) and prompted cell cycle arrest in the G2/M phase for various cell lines (Fig. 2f, g). Further validation in vivo through subcutaneous injection of SW620 cells into BALB/c nude mice demonstrated the ability of TMPyP4 to attenuate tumor growth, reflected in markedly lower tumor weights and volumes in the TMPyP4 treatment group compared to controls (Fig. 2h, i). This effect was consistent in a CRC patient-derived xenograft (PDX) model, where TMPyP4 successfully suppressed tumor growth (Fig. 2j, k). These results suggest that TMPyP4-induced G4 structure formation contributes to impairing cancer cell proliferation, cell cycle progression, and overall survival.

### G4 ligand enhanced the antitumor immune effect in vivo

**Fig. 3:**
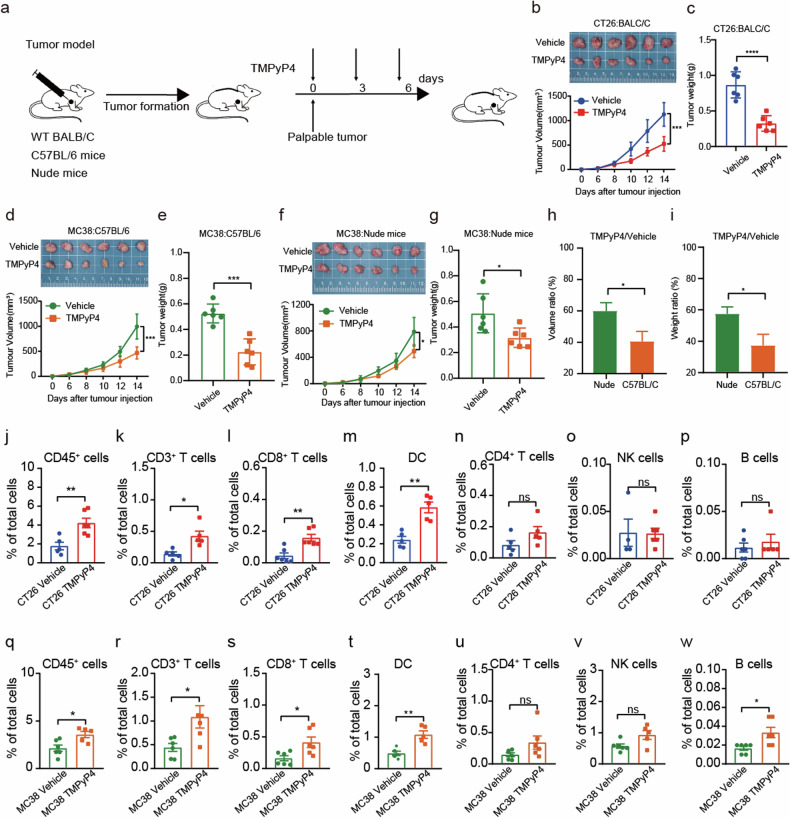
G4 ligand TMPyP4 reduces the tumor growth of the subcutaneous colorectal cancer model by increasing CD8^+^ T cells and DCs. **a** Experimental treatment strategy for tumor growth inhibition in a syngeneic mouse tumor model. When tumors were palpable, mice were treated with vehicle or 30 mg/kg TMPyP4 three times a week (days 0, 3, 6). **b**, **c** The tumor size and tumor weight of BALB/C mice bearing CT26 colon cancer treated with vehicle or 30 mg/kg TMPyP4. *n* = 6 mice for both groups. **d**, **e** The tumor size and tumor weight of C57BL/6 mice bearing MC38 colon cancer treated with vehicle or 30 mg/kg TMPyP4. *n* = 6 mice for both groups. (**f**, **g**) The tumor size and tumor weight of nude mice bearing MC38 colon cancer treated with vehicle or 30 mg/kg TMPyP4. *n* = 6 mice for both groups. **h, i** Tumor growth inhibition comparison between C57BL/6 and nude mice after TMPyP4 treatment. **j**–**p** Graphs show the frequencies of CD45^+^ cells (**j**), CD3^+^ T cells (**k**), CD8^+^ T cells (**l**), DCs (**m**), CD4^+^ T cells (**n**), NK cells (**o**), and B cells (**p**) in CT26 tumors after TMPyP4 treatment or vehicle control treatment. **q**–**w** Graphs show the frequencies of CD45^+^ cells (**q**), CD3^+^ T cells (**r**), CD8^+^ T cells (**s**), DCs (**t**), CD4^+^ T cells (**u**), NK cells (**v**), and B cells (**w**) in MC38 tumors after TMPyP4 treatment or vehicle control treatment. *ns*: not significant, ^*^p ≤ 0.05, ^***^p ≤ 0.001, ^****^p ≤ 0.0001, by two-way ANOVA (**b**, **d**, **f**), or by untailed t-tests (**c**, **e**, **g**–**w**).

To investigate the enhancement of the antitumor immune effect of TMPyP4, we employ an immunocompetent mice subcutaneous tumor-bearing model. CT26 and MC38 cells were subcutaneously injected into syngeneic mice, and upon the palpability of tumors (1-week post-injection), mice were treated intraperitoneally with TMPyP4 (30 mg/kg) or vehicle control three times weekly. Tumor growth and animal body weight were meticulously monitored throughout the study (Fig. 3a). Notably, TMPyP4-treated mice exhibited a significant suppression in tumor growth compared to the vehicle-treated group (Fig. 3b–e). In addition, the biosafety of TMPyP4 has been proved in this study (Supplementary Fig. 3a, b, Supplemental Table S3-4). We extended our evaluation to immune-compromised mice using MC38 cells. TMPyP4 administration, while delaying tumor growth in treated mice compared to the vehicle control (Fig. 3f, g), resulted in a less pronounced inhibition of tumor growth in immunocompromised mice compared to immune-competent C57/BL6 mice (Fig. 3h, i). These collective findings suggest a potential dual mode of action for TMPyP4 involving the blockade of cancer cell proliferation and modulation of the immune system.

### TMPyP4 activates tumor immune responses by enhancing the activation of CD8^+^ T cells and maturation of DCs

**Fig. 4:**
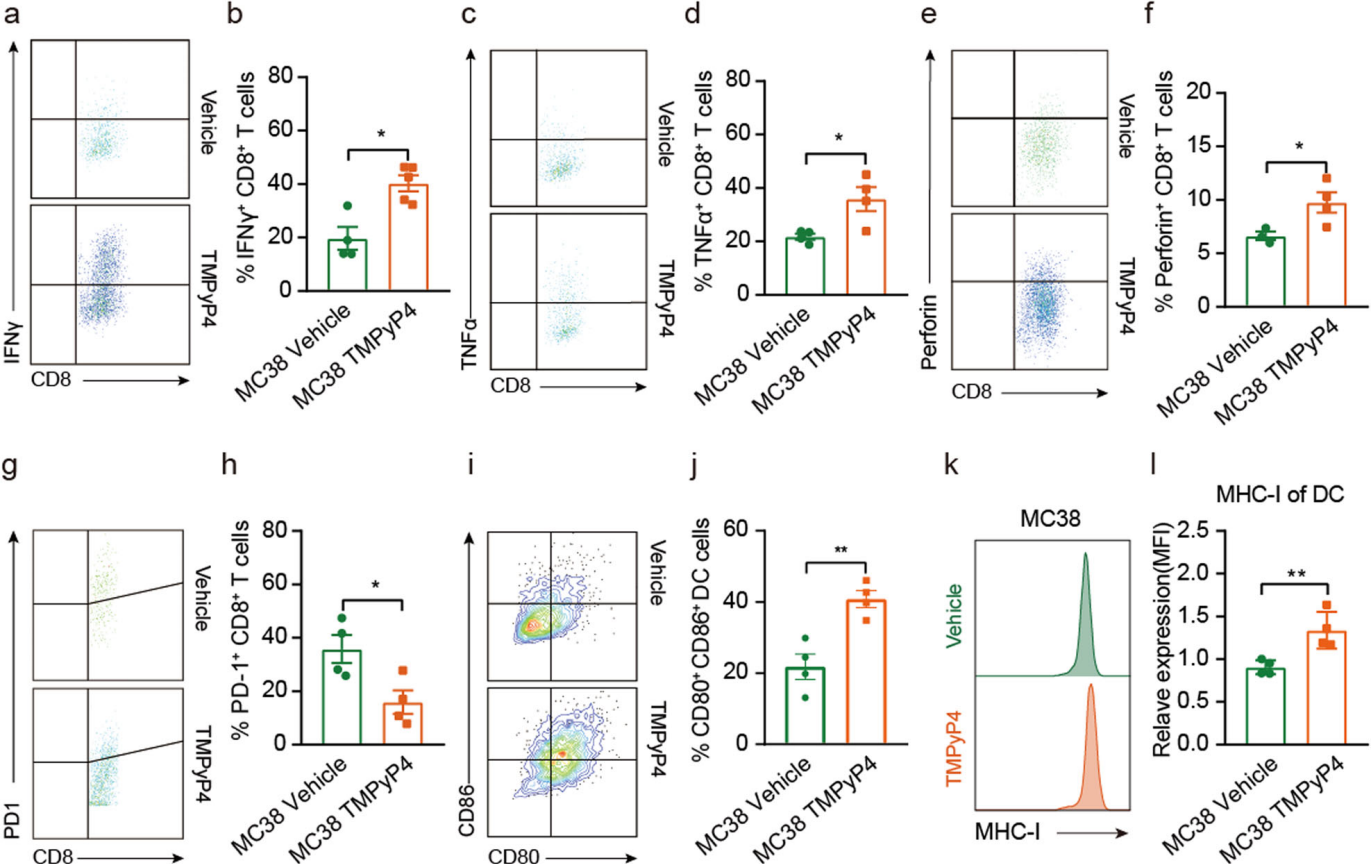
G4 ligand TMPyP4 promotes the activation of CD8^+^ T cells and the maturation of DCs. **a**–**h** Representative FACS plots and quantification of IFNγ expression (**a**, **b**), TNFα expression (**c**, **d**), perforin expression (**e**, **f**), and PD-1 expression (**g**, **h**) among CD8^+^ T cells in MC38 tumors with TMPyP4 treatment or vehicle control treatment. **i**–**l** Surface expression of CD80, CD86, and MHC-I on DCs was determined by FACS. Values are represented as mean ± SD, ^*^p ≤ 0.05, ^**^p ≤ 0.01, ^****^p ≤ 0.0001, by untailed t-tests (**b**, **d**, **f**, **h**, **l**).

To delve into the immune modulatory effects of TMPyP4, we conducted a detailed investigation by employing multicolor flow cytometry to determine its impact on tumor-infiltrating immune cells. Supplementary Fig. 4a provides the gating strategy for identifying immune cells. Our flow cytometry analysis of tumor-infiltrating immune cells from TMPyP4-treated mice revealed an increase in the frequencies of CD45^+^, CD3^+^, CD8^+^ T cells, and DCs (Fig. 3j–m, q–t). In contrast, CD4^+^ T cells and natural killer (NK) cells were minimally affected (Fig. 3n-o, u, v). While tumor-infiltrating B cells tended to increase in MC38 tumors treated with TMPyP4, no significant differences were observed in CT26 tumors (Fig. 3p, w). We then assessed the ability of TMPyP4 to activate CD8^+^ T cells and DCs. TMPyP4 therapy significantly increased the percentages of IFNγ^+^, TNFα^+^, and Perforin^+^ CD8^+^ T cells in the tumor microenvironment compared to control groups (Fig. 4a–f). Furthermore, tumor-infiltrating PD-1^+^ exhausted CD8^+^ T cells were significantly decreased in MC38 tumors treated with TMPyP4 (Fig. 4g, h). The surface expression of activation markers, including CD80, CD86, and MHC-I on tumor-infiltrating DCs, was markedly increased in TMPyP4-treated MC38 tumors (Fig. 4i–l). These data suggest that TMPyP4 can induce potent anti-tumor immunity by enhancing the activation of CD8^+^ T cells and the maturation of DCs.

### STING-dependent anti-tumor immune mechanism responsible for tumor suppression by TMPyP4

**Fig. 5:**
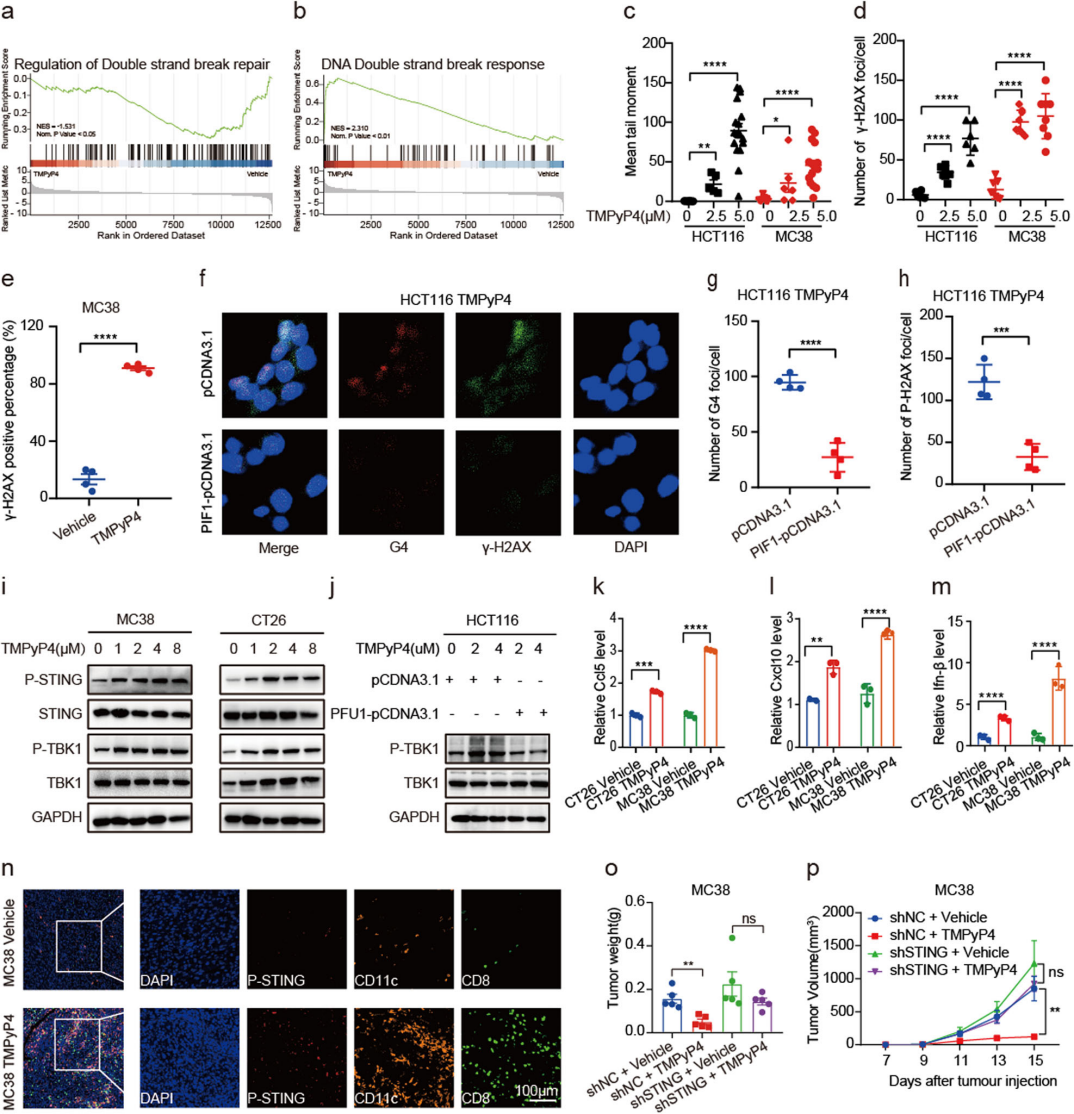
TMPyP4 induces DNA damage in a G4-dependent manner and activates the cGAS-STING pathway. **a**, **b** GSEA of the signaling pathway, including regulation of double-strand break repair and DNA double-strand break response in MC38 tumors with TMPyP4 treatment or vehicle control treatment. **c** Mean tail moment of the HCT116 and MC38 cells treated with or without TMPyP4 in a comet assay. **d** Quantification of foci/nucleus of γ-H2AX immunofluorescence staining of HCT116 and MC38 cells treated with or without TMPyP4. **e** Statistical analysis of γ-H2AX in MC38 tumors with TMPyP4 treatment or vehicle control treatment. **f** Representative images of G4 and γ-H2AX immunofluorescence staining of HCT116 treated with plasmids pCDNA3.1 or PIF1-pCDNA3.1 in the presence of TMPyP4. **g**, **h** Quantification of foci/nucleus of G4 and γ-H2AX in (**f**). **i**, **j** Western blots of p-TBK1, TBK1, p-STING, STING, and GAPDH in colorectal cancer cells with the indicated concentrations of TMPyP4. **k**–**m** RNA levels of Ccl5, Cxcl10, and Ifn-β were detected in CT26 and MC38 treated with or without TMPyP4 for 48 h. **n** Representative immunostainings of CD8, CD11c, and P-STING in paraffin sections of MC38 tumors with TMPyP4 treatment or vehicle control treatment (CD8 [green], CD11c [orange], P-STING [red], DAPI [blue]) (magnification ×200). **o**, **p** The tumor size and tumor weight of C57BL/6 mice bearing indicated MC38 colon cancer treated with vehicle or 30 mg/kg TMPyP4. *n* = 5 mice for both groups. Values are represented as mean ± SD, *ns*: not significant, ^**^p ≤ 0.01, ^***^p ≤ 0.001, ^****^p ≤ 0.0001, by untailed t-tests (**c**, **d**, **e**, **g**, **h**, **k**, **l**, **m**), or by two-way ANOVA (**g**).

To identify the key signaling pathways regulated by TMPyP4, we conducted RNA sequencing on CRC cell lines MC38, treated with either vehicle or TMPyP4. Intriguingly, TMPyP4-regulated targets were enriched in functional clusters, especially those related to the cell cycle and immune response, suggesting pivotal roles of TMPyP4 in tumor cell proliferation and immune regulation (Supplementary Fig. 4b–e). Further analysis through gene set enrichment analysis (GSEA) implied a potential impact of TMPyP4 on CRC progression through DNA damage (Fig. 5a, b), which was consistent with the previous study of the G4 stabilization-induced DNA damage [21]. To validate this, we performed the comet assay, revealing a substantial increase in comet tail length in HCT116 and MC38 cell lines following TMPyP4 treatment (Fig. 5c, Supplementary Fig. 4f). Additionally, examination of γH2AX levels, a marker for dsDNA breaks, showed a dose-dependent increase in HCT116 and MC38 cell lines treated with TMPyP4 (Fig. 5d, Supplementary Fig. 4g). We also confirmed the induction of DNA damage in vivo by TMPyP4 treatment using IHC (Fig. 5e, Supplementary Fig. 4h). To further confirm that TMPyP4 induces DNA damage depending on the G4 structure, we found that the PIF1 DNA helicase, a potent unwinder of G4 structures [22], can counteract the DNA damage induced by TMPyP4 (Fig. 5f–h). These findings suggest that TMPyP4 induces DNA damage in a G4-dependent manner.

Recent studies have implicated the cGAS-STING pathway in detecting cytosolic DNA and activating immune responses upon DNA damage [23]. Given that TMPyP4 increased DNA damage in colorectal cancer cells, we hypothesized that TMPyP4 might enhance the immune response through the cGAS-STING pathway. Supporting this, the protein levels of phosphorylated TANK-binding kinase 1 (TBK1) and stimulator of interferon genes (STING) were elevated in the TMPyP4 treatment group compared to the vehicle control, while the total expression of TBK1 and STING remained unchanged. However, the G4 helicase PIF1 can nullify this effect (Fig. 5i, j, Supplementary Fig. 4i). Additionally, mRNA levels of cytokines (Ccl5, Cxcl10, and Ifn-β) were elevated in the TMPyP4 treatment group compared to the vehicle control (Fig. 5k–m). This notion is further supported by the increased expression of P-STING, CD8^+^ T cells, and DCs observed via mIHC following TMPyP4 treatment in vivo (Fig. 5n). To directly confirm the role of cGAS-STING in TMPyP4-induced anti-tumor immunity, colon cancer cells with or without STING knockdown were treated with or without TMPyP4. The efficiency of STING knockdown is shown in Supplementary Fig. 5j. Notably, TMPyP4 did not impede tumor progression in STING-knockdown MC38 cells (Fig. 5o, p). These data highlight that TMPyP4-induced DNA damage may activate the cGAS-STING signaling pathway, contributing to enhanced antitumor immunity.

### TMPyP4 enhances immunotherapeutic response

**Fig. 6:**
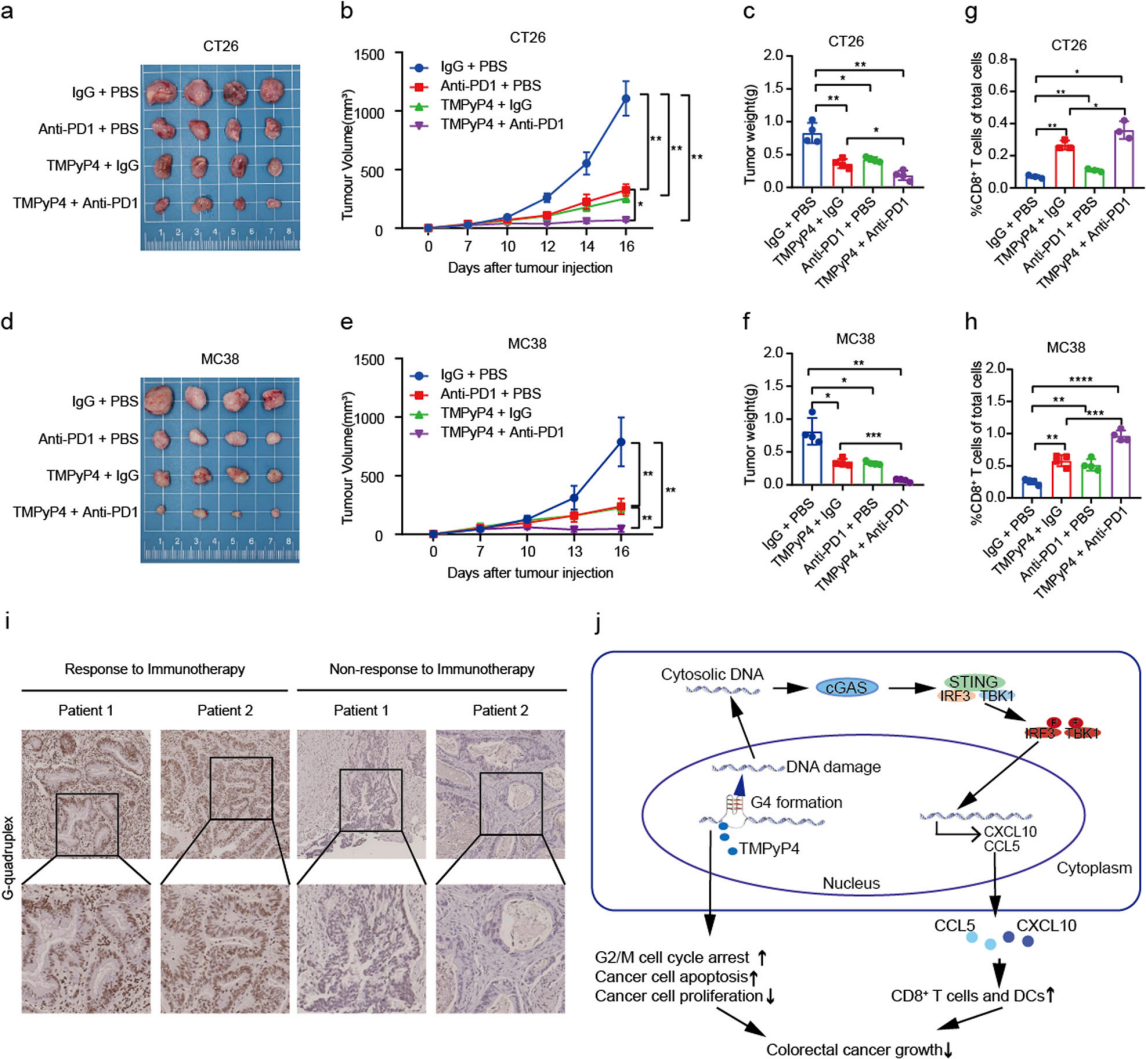
G4 ligand TMPyP4 enhances antitumor responses induced by anti-PD1. **a**–**f** Tumor growth of vehicle, TMPyP4 alone (30 mg/kg), anti-PD1 alone (200 μg/body), and TMPyP4 plus anti-PD1 groups in BALB/C mice injected with CT26 cells (**a**–**c**) or C57BL/C mice injected with MC38 cells (**d**–**f**). *n* = 4 mice for each group. **g**, **h** The frequencies of CD8^+^ T cells in CT26 (**g**) and MC38 (**h**) tumors after vehicle, TMPyP4 alone (30 mg/kg), anti-PD1 alone (200 μg/body), and TMPyP4 plus anti-PD1 groups treatment. Values are represented as mean ± SD, *p ≤ 0.05, **p ≤ 0.01, ***p ≤ 0.001, by two-way ANOVA (**b**, **e**), or by untailed t-tests (**c**, **f**, **g**, **h**). **i** Relationship between G4 expression and immunotherapy efficacy in colorectal patients. **j** The overview of the study.

In roder to clarify whether the formation of the G4 structure by TMPyP4 contributes to enhanced antitumor immunity, we subcutaneously transplanted murine colon cancer CT26 and MC38 cells into immunocompetent mice, and treated them with anti-PD1 once the xenografts became palpable. Notably, the TMPyP4 treatment group exhibited heightened sensitivity to anti-PD1 treatment compared to mice treated with the vehicle control (Fig. 6a–f). Consistent with this, there was an increase in the number of CD8^+^ T cells in the TMPyP4 treatment group (Fig. 6g, h). These findings strongly suggest that TMPyP4 promotes antitumor immunity. To further assess the relevance in human tumors, we conducted IHC to measure the expression of G4 in patients undergoing immunotherapy. Our analyses revealed that patients with high G4 structure expression were more responsive to immunotherapy, proposing G4 as a novel determinant influencing the clinical response to immunotherapy (Fig. 6i). Overall, these data underscore the remarkable potential of G4 in augmenting immune therapy against CRC.

## Discussion

Colorectal cancer is the third most common malignant tumor globally, posing a significant societal burden [24]. Recent breakthroughs in immunotherapy targeting the PD-1/PD-L1 pathway have shown promise [25]. However, the challenge persists in achieving a robust response in a limited subset of patients. Enhancing this response is a critical objective in improving colorectal cancer treatment outcomes.

Our study unveils the G4 ligand TMPyP4 as a potent activator of the cGAS-STING pathway. This activation cascades into increased CD8^+^ T cell activation and dendritic cell maturation, ultimately enhancing the efficacy of immunotherapy. By shedding light on this crucial mechanism, our findings suggest that TMPyP4 is a potential target for combination immunotherapy strategies involving PD-1/PD-L1 checkpoint blockade.

Guanine-rich DNA sequences can form G4, non-canonical four-stranded secondary structures with established roles in fundamental biological processes [13]. The involvement of G4 in DNA replication, transcription, genomic stability, and epigenetic regulation is well-established [26–28]. These structures have been extensively studied in various diseases, including cancer, highlighting their significance as potential therapeutic targets [29].

TMPyP4, a G4 ligand, has been recognized for stabilizing G4 structures in vitro and in vivo [30, 31]. Its selective concentration in tumor tissues enhances its effectiveness in targeting cancer cells. While previous studies have highlighted the advantages of TMPyP4 in osteosarcoma treatment, but its role in colorectal cancer has not been extensively explored [32]. Our study demonstrates the potent impact of TMPyP4 in inhibiting cancer progression and eliciting a robust anti-tumor immune response. As a G4 ligand, TMPyP4 induces DNA damage and activates the cGAS-STING pathway. This activation, in turn, contributes to the activation of CD8^+^ T cells and the maturation of DCs, emphasizing the multifaceted role of TMPyP4 in modulating the tumor microenvironment.

The limited effectiveness of anti-PD1/PD-L1 therapy often stems from insufficient CD8^+^ T lymphocyte infiltration, hampering the immune system’s ability to recognize and respond to cancer cells [33, 34]. The ability of TMPyP4 to enhance anti-PD1 efficacy by promoting CD8^+^ T cell activation positions it as a novel therapeutic target. Our findings underscore potential of TMPyP4 to overcome hurdles in immunotherapy for colorectal cancer, making it a promising avenue for future investigation.

In this study, we present a novel immune activation mechanism, highlighting the unique ability of TMPyP4 to activate the cGAS-STING pathway, enhancing CD8^+^ T cell activation and dendritic cell maturation. This offers a distinct advantage in anti-tumor immunity compared to conventional CRC therapies. Furthermore, we observe significant synergy between TMPyP4 and anti-PD1 immunotherapy, particularly beneficial for CRC patients with microsatellite-stable (MSS) tumors, who typically exhibit limited responses to immunotherapy alone. The selective stabilization of G4 structures by TMPyP4 may mitigate the off-target effects and toxicity often associated with standard chemotherapy, presenting it as a promising adjunct treatment. Additionally, TMPyP4 may help overcome drug resistance linked to conventional therapies. Its G4-targeting mechanism could facilitate a more personalized treatment strategy for patients, contrasting the one-size-fits-all approach of traditional treatments.

## Supplementary information

Supplementary Figure

Supplementary table

Supplementary material

## Data Availability

The datasets generated during and/or analyzed during the current study are available from the corresponding author upon reasonable request. The transcriptomic data can be acquired in Gene Expression Omnibus (GEO) datasets (https://www.ncbi.nlm.nih.gov/geo/) with accession number GSE280105.
