# Supplementary material for: Targeting G-quadruplex by TMPyP4 for inhibition of colorectal cancer through cell cycle arrest and boosting anti-tumor immunity
Source: Cell Death Dis. 2024 Nov 11;15(11):816. doi: 10.1038/s41419-024-07215-2 (PMC11554887; doi:10.1038/s41419-024-07215-2)
Supplement: Supplementary file 1 — Supplementary Figure [file 41419_2024_7215_MOESM1_ESM.docx]

**Supplementary figures**


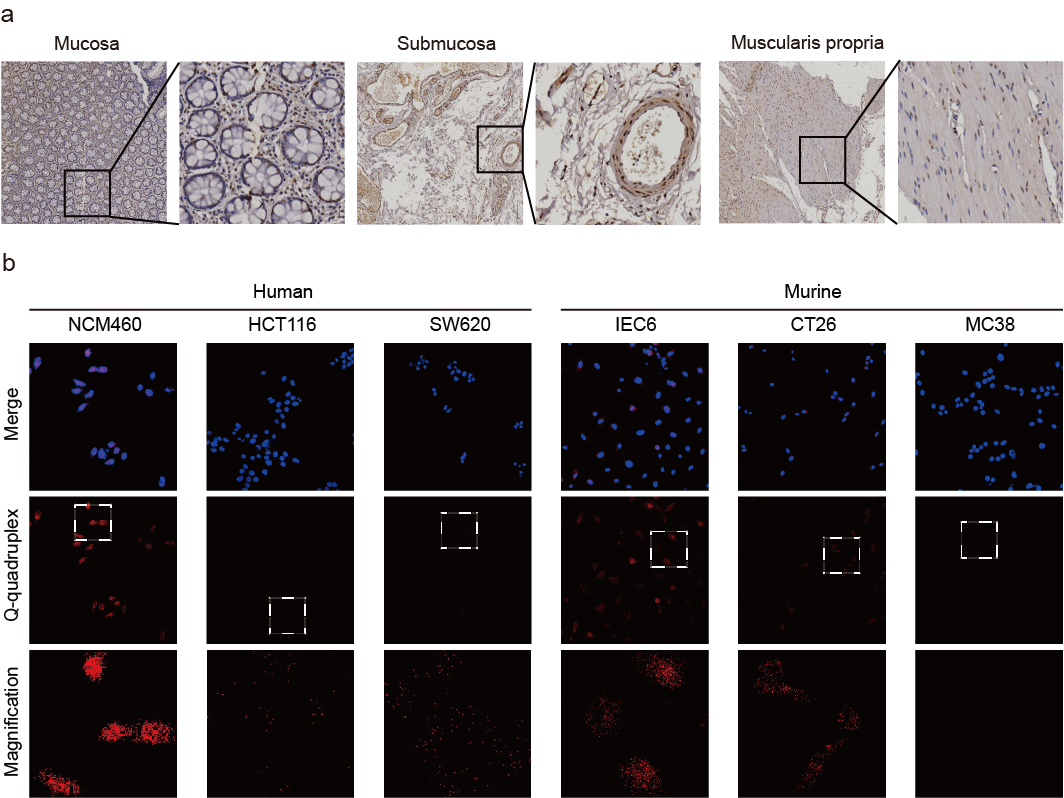


**Supplementary Fig 1.** **(a)** Representative immunostainings of G4 across all layers of normal tissue, including the mucosa, submucosa, and muscularis propria. **(b)** Immunofluorescence staining for G4 in human normal epithelial cell NCM460, colorectal cancer cells HCT116, SW620, murine normal epithelial cell IEC6, colon cancer cells CT26, and MC38.

**
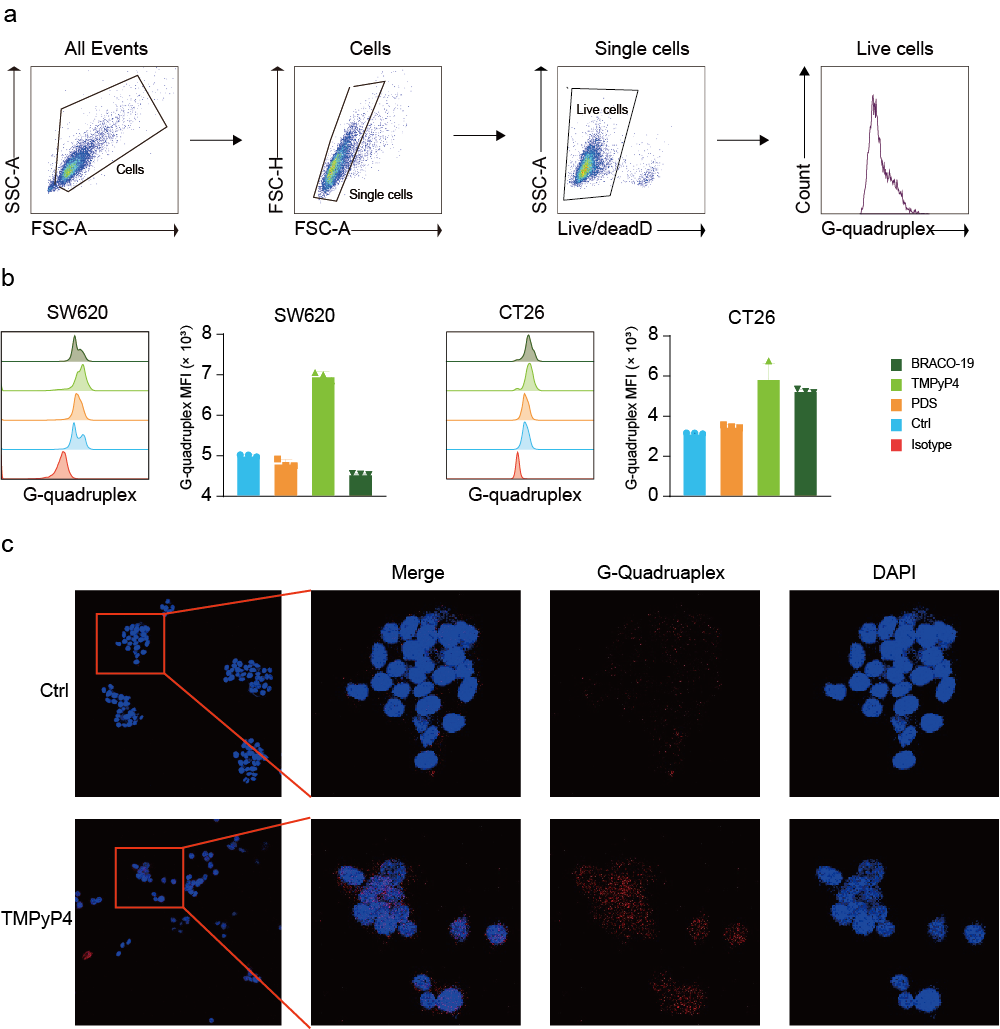
Supplementary Fig 2. (a)** Gating strategy for G4 staining by flow cytometry. **(b)** Flow cytometric analysis for G4 in SW620 and CT26 treated with 10μM PDS, 5μM BRACO-19c, and 5μM TMPyP4 for 12h. **(c)** Immunofluorescence staining for G4 in SW620 treated with or without 5μM TMPyP4 for 12h.


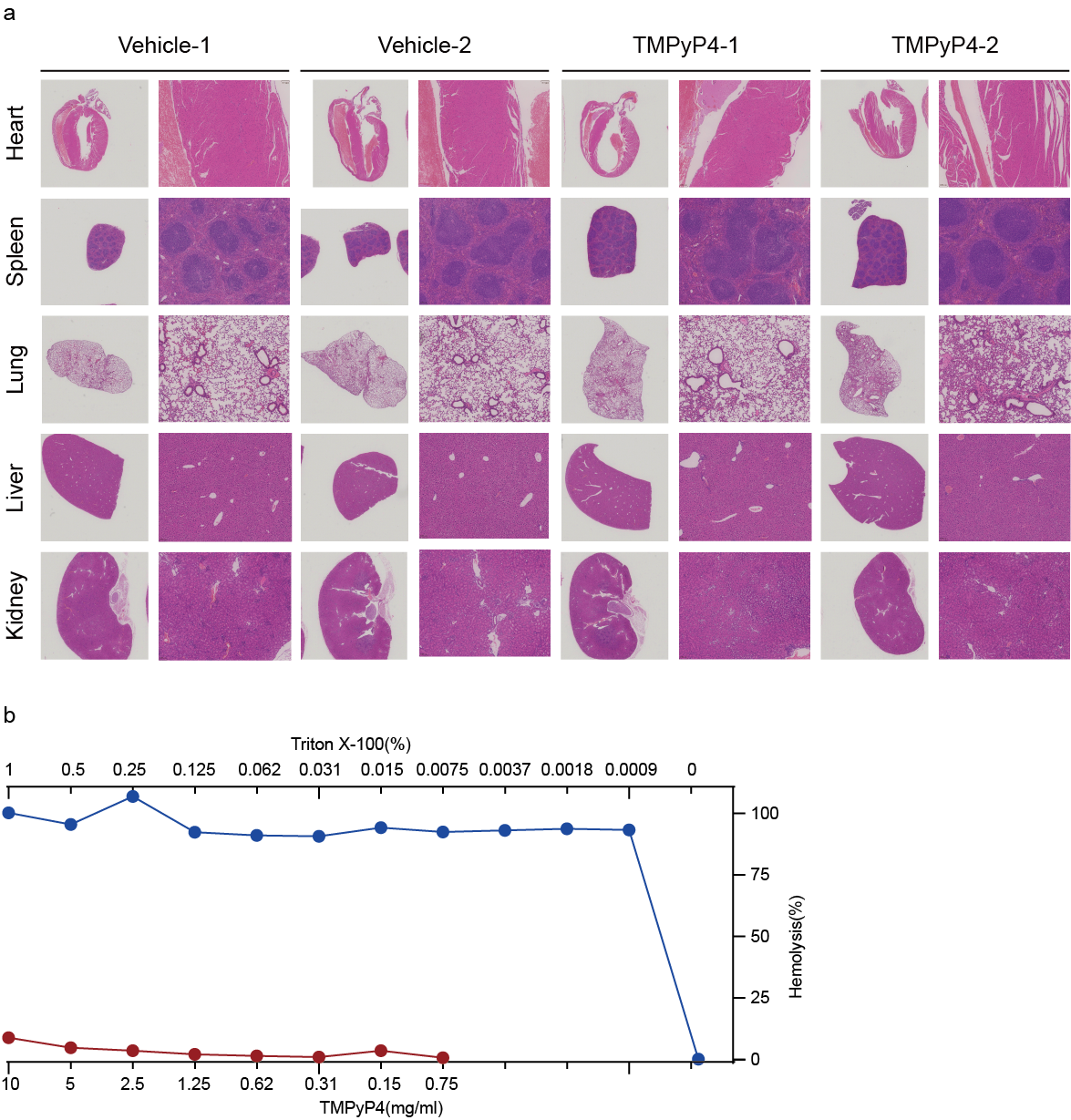
**Supplementary Fig 3.** **(a)**Representative images of heart, liver, spleen, lung, and kidney of C57BL/6 mice treated with vehicle or 30 mg/kg TMPyP4. **(b)** Percentage of hemolysis induced by the indicated concentrations of TMPyP4. Triton-X-100 was used as positive control.


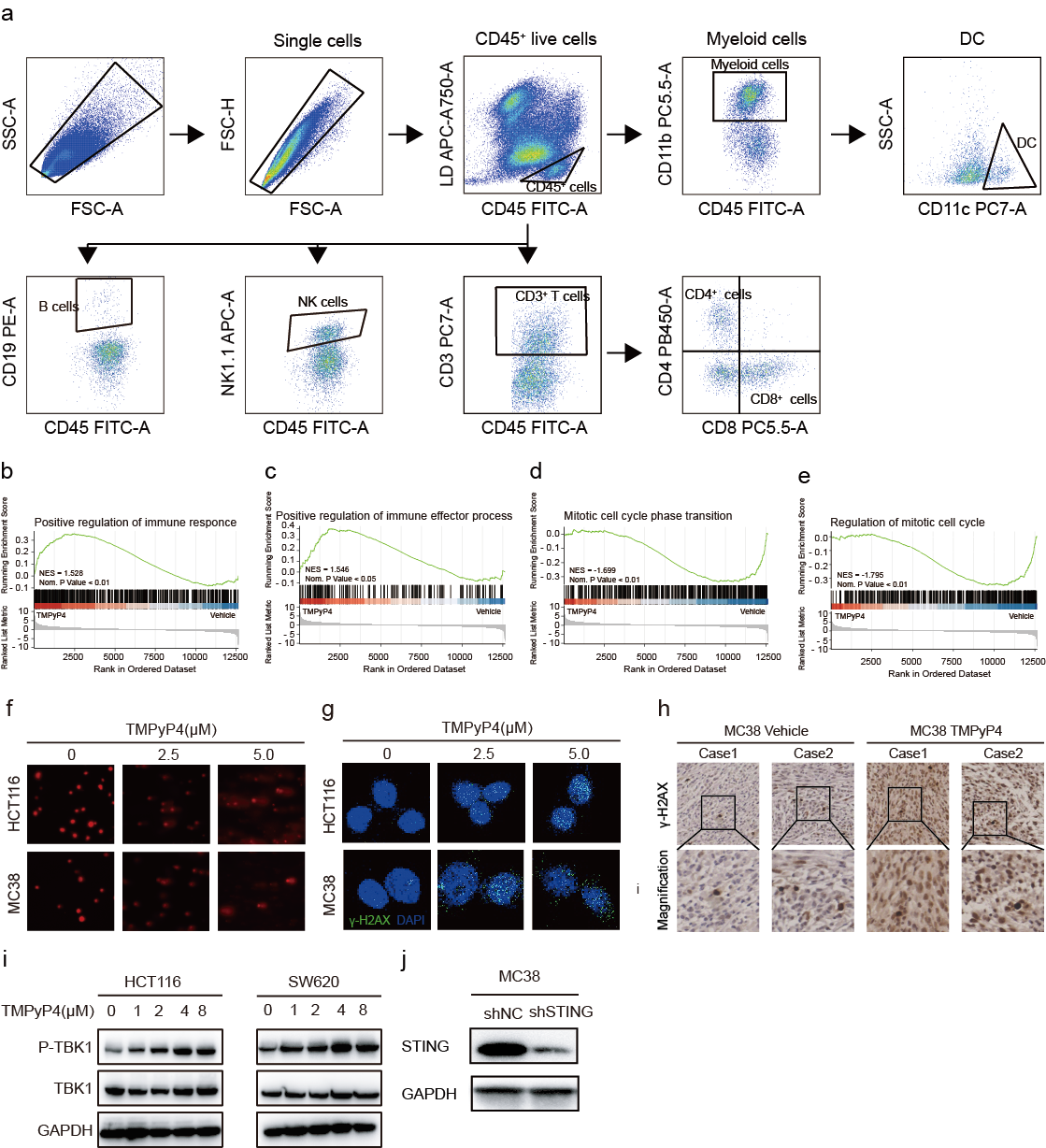


**Supplementary Fig 4.** **(a)** Gating strategy for immune cells population and additional data on intratumoral number of immune cells. Live cells were selected by the live/dead dye. CD45^+^NK1.1^+^ cells were defined as NK cells. CD45^+^CD19^+^ cells were defined as B cells. CD8^+^ and CD4^+^ T lymphocytes were from the CD45^+^CD3^+^CD8^+^ and CD45^+^CD3^+^CD4^+^ subpopulation respectively. DC were defined as the CD45^+^CD11b^+^CD11c^+^ subset. **(b-e)** GSEA of signaling pathway, including regulation of positive regulation of immune response **(b)**, positive regulation of immune effector process **(c)**, mitotic cell cycle phase transition **(d)**, and regulation of mitotic cell cycle **(e)** in MC38 tumors with TMPyP4 treatment or vehicle control treatment. **(f)** Comet images of the HCT116 and MC38 cells treated with or without TMPyP4 for 24 h, stained with Protech DNA dye. **(g)** Representative images of foci/nucleus of γ-H2AX immunofluorescence staining of HCT116 and MC38 cells treated with or without TMPyP4 for 24 h. **(h)** Representative images of γ-H2AX IHC staining in MC38 tumors with TMPyP4 treatment or vehicle control treatment. **(i)** Western blots of p-TBK1, TBK1, and GAPDH in colorectal cancer cells HCT116 and SW620 with the indicated concentrations of TMPyP4. **(j)** Western blots of MC38 transfected with plasmid shRNA targeting STING with STING and GAPDH antibody.
