# Supplementary material for: Targeting G-quadruplex by TMPyP4 for inhibition of colorectal cancer through cell cycle arrest and boosting anti-tumor immunity
Source: Cell Death Dis. 2024 Nov 11;15(11):816. doi: 10.1038/s41419-024-07215-2 (PMC11554887; doi:10.1038/s41419-024-07215-2)
Supplement: Supplementary file 2 — Supplementary table [file 41419_2024_7215_MOESM2_ESM.docx]

**Supplementary Table S1. Information of antibodies**

| Antibody | Source | Identifier |
| --- | --- | --- |
| Anti-Cytokeratin Pan Type I/II | Invitrogen | Cat# MA1-82041 |
| Anti-CD8 (C8/144B) | Dako | Code# M7103 |
| Anti-DNA G-quadruplex (1H6) | Merck | Cat# MABE1126 |
| Anti-CD45 | BioLegend | Cat# 103108 |
| Anti-CD3e (145-2C11) | Invitrogen | Cat# 25-0031-82 |
| Anti-CD8a (53-6.7) | Invitrogen | Cat# 45-0081-82 |
| Anti-CD4 (GK1.5) | Invitrogen | Cat# 48-0041-82 |
| Fixable Viability Dye | Invitrogen | Cat# 65-0865-14 |
| Anti-NK1.1 (PK136) | Invitrogen | Cat# 17-5941-81 |
| Anti-F4/80 | BioLegend | Cat# 123115 |
| Anti-CD19 | BioLegend | Cat# 115507 |
| Anti-Perforin (eBioOMAK-D) | Invitrogen | Cat# 12-9392-82 |
| Anti-CD279 (J43) | Invitrogen | Cat# 12-9985-82 |
| Anti-IFN-γ | BioLegend | Cat# 505808 |
| Anti-TNF alpha (MP6-XT22) | Invitrogen | Cat# 17-7321-81 |
| Anti-CD11c | BioLegend | Cat# 117317 |
| Anti-CD11b (M1/70) | Invitrogen | Cat# 45-0112-80 |
| Anti-MHC Class I (H-2Kb) | eBioscience | Cat# 17-5958-80 |
| Anti-CD86 | Invitrogen | Cat#12-0862-82 |
| Anti-CD80 | Invitrogen | Cat#11-0801-82 |
| Anti-CD45 | BioLegend | Cat# 103155 |
| Anti-Phospho-TBK1/NAK (Ser172) | Cell Signaling Technology | Cat# 5483 |
| Anti-TBK1/NAK (D1B4) | Cell Signaling Technology | Cat# 3504 |
| Anti-Phospho-Histone H2A.X (Ser139) | Merck | Cat# 05-636-25 |
| Anti-Phospho-STING (Ser365) (D8F4W) | Cell Signaling Technology | Cat # 72971 |
| Anti-STING (E9X7F) | Cell Signaling Technology | Cat # 90947 |
| Anti-CD8 (D8A8Y) | Cell Signaling Technology | Cat # 85336 |
| Anti-CD11c (D1V9Y) | Cell Signaling Technology | Cat # 97585 |
| Anti-PD1 (RMP1-14) | InvivoMab | Cat # BE0146 |
| Anti-IgG2a (2A3) | InvivoMab | Cat # BE0089 |

**Supplementary Table S2. Primers and DNA sequences used.**

| Name | Sequence | Application |
| --- | --- | --- |
| Cxcl10-forward | CCAAGTGCTGCCGTCATTTTC | qRT-PCR |
| Cxcl10-reverse | GGCTCGCAGGGATGATTTCAA | qRT-PCR |
| Ccl5-forward | TTTGCCTACCTCTCCCTCG | qRT-PCR |
| Ccl5-reverse | CGACTGCAAGATTGGAGCACT | qRT-PCR |
| Ifn-β-forward | TCCGAGCAGAGATCTTCAGGAA | qRT-PCR |
| Ifn-β-reverse | TGCAACCACCACTCATTCTGAG | qRT-PCR |
| sh-STING | CCGGCAACATTCGATTCCGAGATATCTCGAGATATCTCGGAATCGAATGTTGTTTTTGAATT | shRNA target site |

**Supplementary Table S3. Serum biochemical parameters of mice**

| **NO.** | **Index** | **Radius** | **TMPyP4（mg/kg）** | |
| --- | --- | --- | --- | --- |
|  |  |  | **0 (n=2)** | **30 (n=2)** |
| 1 | ALT | 10.06-96.47 U/L | 43.95 ± 2.93 | 46.39 ±1.34 |
| 2 | AST | 36.31-235.48 U/L | 193.66 ± 6.95 | 220.95 ± 20.79 |
| 3 | BUN | 10.81-34.74 mg/dL | 28.508 ± 1.00 | 27.71 ± 0.58 |
| 4 | UA | 44.42-224.77 μmol/L | 155.25 ± 8.47 | 170.11 ± 1.76 |
| 5 | CK | 0-2070.55 U/L | 1674.12 ± 254.00 | 1680.01 ± 319.79 |

Data are presented as means ± SEM. Significant results were determined by the One-way ANOVA with Tukey's post-hoc test. **p*<0.05, ***p*<0.01.

ALT, alanine aminotransferase; AST, aspartate aminotransferase; BUN, blood urea nitrogen; UA, urine acid; CK, creatine kinase.

**Supplementary Table S4. Blood routine test results of mice**

| **NO.** | **Index** | **Radius** | **TMPyP4（mg/kg）** | |
| --- | --- | --- | --- | --- |
|  |  |  | **0 (n=5)** | **30 (n=5)** |
| 1 | WBC | 0.80 - 10.60 | 5.42±1 | 7.35±0.55 |
| 2 | Neu# | 0.23 - 3.60 | 0.38±0.06 | 0.42±0.03 |
| 3 | Lym# | 0.60 - 8.90 | 4.74±0.95 | 6.58±0.55 |
| 4 | Mon# | 0.04 - 1.40 | 0.06±0.01 | 0.07±0 |
| 5 | Eos# | 0.00 - 0.51 | 0.24±0.03 | 0.27±0.02 |
| 6 | Bas# | 0.00 - 0.12 | 0±0 | 0.01±0 |
| 7 | Neu% | 6.5 - 50.0 | 7.12±0.9 | 5.94±0.79 |
| 8 | Lym% | 40.0 - 92.0 | 86.74±1.77 | 89.24±0.99 |
| 9 | Mon% | 0.9 - 18.0 | 1.12±0.21 | 0.94±0.11 |
| 10 | Eos% | 0.0 - 7.5 | 4.86±0.82 | 3.76±0.24 |
| 11 | Bas% | 0.0 - 1.5 | 0.16±0.02 | 0.12±0.02 |
| 12 | RBC | 6.50 - 11.50 | 6.15±0.58 | 7.14±0.37 |
| 13 | HGB | 110 - 165 | 133.8±9.77 | 152.8±1.71 |
| 14 | HCT | 35.0 - 55.0 | 36.22±2.86 | 42.3±2.97 |
| 15 | MCV | 41.0 - 55.0 | 59.38±1.84 | 59±1.31 |
| 16 | MCH | 13.0 - 18.0 | 22.04±0.98 | 21.68±1.22 |
| 17 | MCHC | 300 - 360 | 372.8±17.6 | 370±29.52 |
| 18 | RDW-CV | 12.0 - 19.0 | 20.46±0.89 | 20.38±1.28 |
| 19 | RDW-SD | 23.0 - 39.0 | 47.72±1.42 | 47.14±2.15 |
| 20 | PLT | 400 - 1600 | 1449.8±98.15 | 1524.2±46.17 |
| 21 | MPV | 4.0 - 6.2 | 8.02±0.41 | 8.34±0.12 |
| 22 | PDW | 12.0 - 17.5 | 17.84±0.31 | 17.24±0.17 |

Data are presented as means ± SEM. Significant results were determined by the One-way ANOVA with Tukey's post-hoc test. *p<0.05, **p<0.01.

WBC, white blood cell; Lym, lymphocytes; Mon, monocytes; Neu, neutrophils; Eos, eosinophils; Bas, basophils; RBC, red blood cell count; HGB, hemoglobin content; HCT, hematocrit; MCV, mean red blood cell volume; MCH, mean red blood cell hemoglobin; MCHC, mean red blood cell hemoglobin concentration; RDW-CV, red blood cell distribution width-CV; RDW-SD, red blood cell distribution width-SD; PLT, platelet count; MPV, mean platelet volume; PDW, Platelet volume distribution width.
