# Supplementary material for: Targeting G-quadruplex by TMPyP4 for inhibition of colorectal cancer through cell cycle arrest and boosting anti-tumor immunity
Source: Cell Death Dis. 2024 Nov 11;15(11):816. doi: 10.1038/s41419-024-07215-2 (PMC11554887; doi:10.1038/s41419-024-07215-2)
Supplement: Supplementary file 3 — Supplementary material [file 41419_2024_7215_MOESM3_ESM.pptx]

## Slide 1
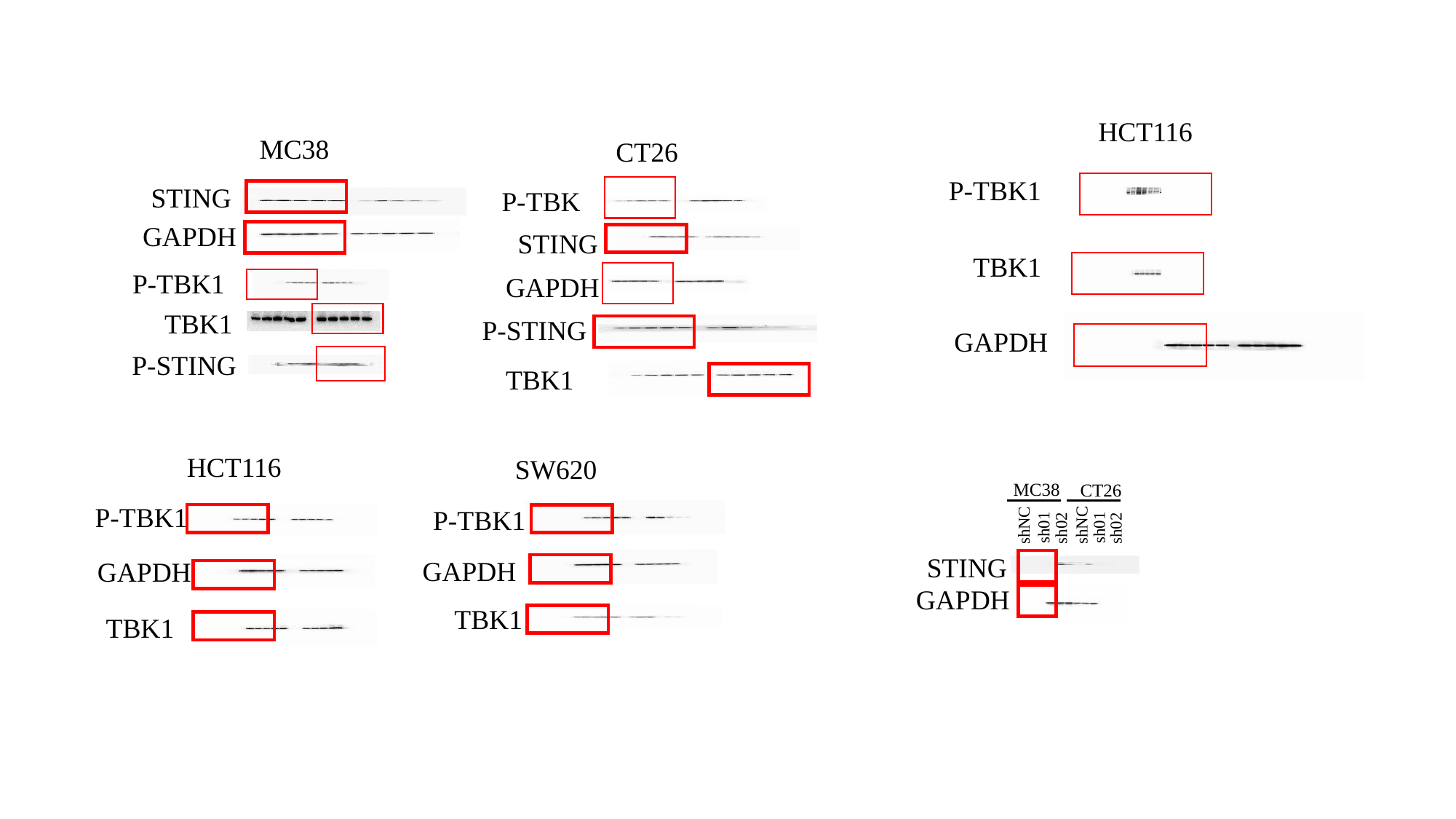

HCT116
MC38
CT26
P-TBK1
STING
P-TBK
GAPDH
STING
TBK1
P-TBK1
GAPDH
TBK1
P-STING
GAPDH
P-STING
TBK1
HCT116
SW620
MC38
CT26
P-TBK1
P-TBK1
shNC
shNC
sh01
sh01
sh02
sh02
STING
GAPDH
GAPDH
GAPDH
TBK1
TBK1
